# Wearable Device Adoption, Physical Activity, and Health Data Sharing Among U.S. Cancer Survivors: Evidence from HINTS-7

**DOI:** 10.3390/jcm15103984

**Published:** 2026-05-21

**Authors:** Zarmina Amin, Jessh Mavoungou, John Oginni, Zan Gao

**Affiliations:** Department of Kinesiology, Recreation, and Sport Studies, The University of Tennessee-Knoxville, 328 HPER Building, 1914 Andy Holt Avenue, Knoxville, TN 37996-2700, USA

**Keywords:** cancer survivorship, digital health, health disparities, physical activity, wearable devices

## Abstract

**Background/Objectives:** Wearable devices are increasingly used to support physical activity (PA), yet national patterns of use and their relationship with PA among cancer survivors remain unclear. Integration of wearable data into clinical care is also poorly understood. This study examined wearable use, its association with meeting PA guidelines, and health data-sharing with providers among U.S. adults with and without cancer. **Methods:** A cross-sectional analysis of the Health Information National Trends Survey (HINTS-7), a nationally representative survey of U.S. adults, was conducted. Survey weights and jackknife replication methods generated population-level estimates. Wearable use (yes/no), meeting PA guidelines (≥150 min/week moderate activity), and data-sharing behaviors were assessed. Weighted logistic regression evaluated associations between wearable use and meeting PA guidelines, including interaction by cancer history. Analyses also examined willingness to share and actual data-sharing. **Results:** The sample included 6084 U.S. adults. Wearable use was lower among cancer survivors (34.0%) than those without cancer (41.4%). Individuals using wearable devices were more likely to meet PA guidelines (ORs: 1.79–1.97), with the association being stronger among cancer survivors. Among cancer-surviving wearable users, willingness to share data with providers was high (77.5%), but actual sharing was substantially lower (35.4%). Few predictors of willingness were identified. **Conclusions:** Wearable use is associated with meeting PA guidelines at the population level, with potential relevance for cancer survivors. However, despite high willingness to share data, clinical integration remains limited, highlighting a gap between digital engagement and healthcare use. Strategies to improve integration of patient-generated data into care are needed.

## 1. Introduction

In the United States (US), more than 18 million individuals are currently living with a history of cancer, and this number is projected to increase substantially in the coming decades [1]. Advances in early detection and treatment have improved survival rates, resulting in a growing population of individuals living beyond a cancer diagnosis [2]. The five-year relative survival rate for all cancers combined has reached approximately 70% in recent years, reflecting meaningful advances in early detection and treatment [3]. However, surviving cancer does not mark the end of health challenges. Many survivors experience long-term physical and psychological consequences related to cancer and its treatment, including fatigue, reduced physical functioning, depression, and increased cardiometabolic risk [4,5]. These ongoing health challenges highlight the need for scalable lifestyle strategies that support long-term health and functional recovery in cancer survivorship.

Physical activity is a modifiable lifestyle behavior positively associated with weight management, cardiorespiratory fitness, fatigue reduction, and psychological health outcomes, including quality of life, among cancer survivors [6,7,8,9,10]. Regular physical activity following a cancer diagnosis has also been associated with reduced cancer recurrence and lower all-cause mortality across multiple cancer types [11,12]. Current guidelines from the American Cancer Society (ACS) recommend that survivors engage in at least 150 min of moderate-intensity aerobic physical activity per week [13]. Despite this evidence, adherence to physical activity among cancer survivors remains poor. In a large cross-sectional study of over 10,000 cancer survivors, only 4% fully adhered to all current ACS lifestyle guidelines, with a mean of just 2.0 guidelines met out of 4.0 [14]. A 2024 national study found approximately half of cancer survivors met aerobic physical activity guidelines, and that adherence was lowest among younger survivors and those with lower income and education [15]. These findings indicate a persistent gap between clinical recommendations and population-level behavior, highlighting the need to better characterize how physical activity is achieved and maintained in survivorship.

Wearable devices, including smartwatches and fitness trackers, are widely used tools for monitoring physical activity and related health behaviors. These devices provide real-time feedback on activity levels, goal tracking, and behavioral metrics that may facilitate self-monitoring [16]. Meta-analytic and systematic review evidence has reported associations between wearable device use and higher physical activity levels and related health outcomes among individuals with cancer [17,18]. However, much of this evidence is derived from intervention studies or selected samples, and nationally representative estimates of wearable use and associated behaviors among cancer survivors remain limited. In the context of cancer survivorship, wearable devices are particularly relevant as tools for monitoring and promoting physical activity, the behavioral metric most directly linked to recurrence risk, mortality, and quality of life outcomes. In addition, it remains unclear whether patterns observed in cancer survivors differ from those in the broader adult population when examined within the same dataset.

Beyond behavioral monitoring, wearable devices generate continuous streams of patient-generated health data that could be relevant to survivorship care. At a population level, less is known about how often these data are shared with healthcare providers and whether willingness to share translates into actual sharing behavior. Prior research has identified barriers to integrating wearable-generated data in clinical care, including challenges related to data standardization, electronic health record integration, and workflow constraints [19]. National survey analyses suggest that many wearable users report willingness to share with providers, yet substantially fewer report having done so [20]. Together, these findings suggest a potential gap between patient willingness and real-world data use, though this gap has not been well quantified using current nationally representative data among cancer survivors.

Despite growing interest in wearable technologies within oncology and digital health, current population-level estimates of wearable adoption, physical activity behaviors, and wearable data-sharing practices among cancer survivors using recent nationally representative data remain limited. Few studies have examined these domains simultaneously within a single dataset or quantified the discrepancy between willingness to share and actual data-sharing behavior. One prior study used the HINTS-5 Cycle 4, 2021 to examine health behavior guideline adherence among survivors from three SEER registries [21]; however, wearable device use and data-sharing behaviors were not examined. Using data from the Health Information National Trends Survey (HINTS-7, 2024), the present study provides updated national estimates of wearable device use and physical activity behaviors, examines whether the association between wearable use and meeting aerobic physical activity guidelines differs by cancer survivorship status, and characterizes patterns of wearable data sharing among cancer survivors. These analyses are intended to describe population-level patterns and establish benchmarks rather than to infer causal relationships.

## 2. Materials and Methods

### 2.1. Data Source and Study Design

Data were obtained from the HINTS-7, a nationally representative cross-sectional survey administered by the U.S. National Cancer Institute to assess health information behaviors among U.S. adults. The target population includes civilian, non-institutionalized adults aged 18 years or older residing in the U.S. HINTS-7 data were collected between 25 March and 16 September 2024, using a mixed-mode design in which respondents completed the survey via mail or web. The HINTS program has been conducted regularly since 2003 to examine the public’s access to and use of cancer-related information across the cancer control continuum, including prevention, diagnosis, treatment, and survivorship.

#### 2.1.1. Sampling and Participants

HINTS-7 used a two-stage stratified sampling design. In the first stage, a stratified random sample of residential mailing addresses was selected from a national address-based sampling frame. In the second stage, one adult per household was selected using the next-birthday method, which randomly identifies the adult whose birthday will occur next.

Addresses were stratified by minority concentration (defined as the proportion of racial/ethnic minority residents per census tract: high vs. low) and geographic location (urban, suburban, and rural) to improve representation of racial/ethnic minority and geographically diverse populations across all regions of the U.S. A total of 36,000 addresses were sampled across the four strata.

Of the sampled households, 7278 completed questionnaires were obtained, resulting in a weighted response rate of 27.3%. Household response rates were calculated using the American Association for Public Opinion Research Response Rate 4 formula, which accounts for households of unknown eligibility. Additional details regarding survey administration, response rate calculations, and sampling procedures are described in the HINTS-7 Methodology Report [22].

#### 2.1.2. Data Collection Procedures

Data collection followed a modified Dillman Tailored Design Method, which included multiple mailings to encourage participation. Households received an initial mailing containing a survey packet and a $2 prepaid incentive, followed by a reminder postcard and up to two additional follow-up mailings for non-respondents. A subsample of non-respondents received an additional mailing with a large incentive to improve response rates. Respondents could complete the survey by returning the paper questionnaire or by completing the web-based survey using a unique access code provided in the mailing materials. Surveys were available in English and Spanish.

#### 2.1.3. Sample Size and Response Rates

The final HINTS-7 dataset included 7278 respondents, of whom 70 were classified as partial completers. Survey completion status was determined based on responses to the core survey sections, where Section A covers health information and technology use and Section B covers general health status and demographics. A questionnaire was classified as complete if at least 80% of the questions in Sections A and B were answered. Questionnaires were classified as partially complete if 50–79% of questions in Sections A and B were answered. Surveys with fewer than 50% of applicable questions completed were considered incomplete and were excluded from the publicly available dataset prior to download by the authors.

#### 2.1.4. Survey Completion and Eligibility

Returned surveys were evaluated for eligibility and completeness prior to inclusion in the final analytic dataset. In HINTS-7, a total of 7585 questionnaires were returned, including 7208 complete surveys and 70 partially completed surveys. Both complete and partial-complete responses were retained for analysis, resulting in the final dataset of 7278 respondents. Questionnaires that were blank, incomplete, or identified as duplicates were excluded during data cleaning.

#### 2.1.5. Analytic Samples

Three analytic samples were used. Sample 1 included all respondents with valid data on cancer survivorship status, wearable device use, physical activity, and covariates (*n* = 6084 for the primary adjusted model) and was used for regression models examining associations with physical activity guideline adherence. Sample 2 included cancer survivors (*n* = 1073 identified; *n* = 1062 with valid wearable status) and was used for descriptive analyses of physical activity behaviors by wearable use. Sample 3 included cancer survivors who reported wearable device use (*n* = 330; *n* = 294 with complete data for regression) and was used for wearable data-sharing analyses.

The full adult sample (Sample 1) was used for all regression models examining associations between wearable use and physical activity, whereas survivor-only samples were used for descriptive and data-sharing analyses.

Analytic sample sizes varied across models due to item-level missingness; complete case analysis was used throughout. Given the modest level of missingness and the use of jackknife replicate survey weights, which partially account for non-response, this approach was deemed appropriate. As a sensitivity check, patterns of missingness were examined and found to be distributed across demographic subgroups without a clear systematic pattern. Nonetheless, the potential for bias due to missing data cannot be fully excluded, and future analyses may consider multiple imputation approaches.

### 2.2. Measures

#### 2.2.1. Cancer Survivorship Status

Cancer survivorship status was determined using HINTS-7 item Q1, which asked respondents, “Have you ever been diagnosed as having cancer?” Respondents who answered “yes” were classified as cancer survivors, while those who answered “no” were classified as non-cancer adults.

#### 2.2.2. Sociodemographic Characteristics

Sociodemographic characteristics included age, sex, educational attainment, and household income. These variables were obtained from the demographic section of the HINTS-7 survey instrument and were included as covariates in multivariable analyses, as prior research has demonstrated associations between these factors and both digital health technology adoption and health behavior engagement [17,18,23].

#### 2.2.3. Health and Functional Status

Several health-related variables were included to account for differences in underlying health status that may influence physical activity participation. Body mass index (BMI) was calculated using self-reported height and weight obtained from HINTS-7 items I6 and I7. General health status was assessed using item I1, “In general, would you say your health is…?” with responses ranging from “poor” to “excellent.” Self-rated confidence in managing one’s health was assessed using item I2, with responses ranging from “not confident at all” to “completely confident.” For each measure, responses were assigned increasing numeric values such that higher scores reflected better general health and greater confidence in health management, respectively. Functional limitations were assessed using items I4c and I4d, which measured mobility limitations and pain-related limitations in daily activities, respectively. Both items used a binary response format (“Yes” or “No”).

#### 2.2.4. Mental Health Disorder

Depression and anxiety disorders were assessed using item I5e of the HINTS-7 survey instrument, which asked whether a doctor or other health professional had ever diagnosed the respondent with depression or an anxiety disorder. Response options included “Yes” and “No,” and were coded as a binary variable (yes vs. no).

#### 2.2.5. Physical Activity

Aerobic physical activity was selected as the primary outcome for this study for two substantive reasons. First, wearable devices, the exposure of interest, are specifically designed and validated to capture aerobic activity through continuous heart rate monitoring, step counts, and movement duration [24]. There is currently no gold standard for objective measurement of resistance training volume or intensity in consumer wearable technology; validation studies have demonstrated that wearable accuracy is substantially reduced during resistance exercise compared to aerobic activity [25], and as such, wearable-derived data cannot reliably index muscle-strengthening behavior in the way it can aerobic activity. Second, HINTS-7 captures aerobic activity in minutes per week, enabling direct binary classification against the established ≥150 min/week threshold from ACS survivorship guidelines [13]. Strength training is assessed only as frequency in days per week with no volume or intensity data, precluding a parallel evidence-based threshold. The American College of Sports Medicine roundtable on exercise in cancer survivors has itself acknowledged that resistance training prescription and measurement in survivorship contexts remains less standardized than aerobic prescription, particularly given the need for symptom-specific and treatment-tailored approaches that vary considerably across individuals [23]. For these reasons, aerobic guideline adherence represents the most appropriate and reliably quantifiable primary outcome given the data available. Strength training frequency is examined descriptively only, and future research using datasets that objectively capture both aerobic and resistance training volume will be well positioned to examine combined guideline adherence as a unified outcome.

#### 2.2.6. Wearable Device Use

Wearable device use was assessed using item B8 of the HINTS-7 survey instrument, which asked, “In the past 12 months, have you used an electronic wearable device to monitor or track your health or activity? For example, a Fitbit, Apple Watch, or Garmin Vivofit.” Response options included “Yes,” “No, not in the past 12 months,” and “I have never used an electronic wearable device.” For analysis, responses were recoded into a binary variable indicating wearable device use (yes vs. no); respondents who selected “I have never used an electronic wearable device” were classified as non-users.

Wearable use frequency was assessed using item B9, which asked, “In the past month, how often did you use an electronic wearable device to track your health?” Responses were recoded into a binary variable (any use vs. no use). Participants who reported using a wearable device at any frequency (every day, almost every day, 1–2 times per week, or less than once per week) were classified as wearable users, while those who reported no use in the past month were classified as non-users.

#### 2.2.7. Wearable Data Sharing

Willingness to share wearable-generated health data with healthcare providers was assessed using item B10, “Would you be willing to share health data from your electronic wearable device with your health care provider?” For analytic purposes, responses were recoded into a binary variable indicating willingness to share wearable-generated health data (“Yes” or “No”).

Actual sharing of wearable-generated health data with healthcare providers was assessed using item B11, which asked, “Have you ever shared health data from your electronic wearable device with your health care provider?” Responses were recorded into a binary variable (“Yes” or “No”).

#### 2.2.8. Data Sharing Predictors

Trust in the healthcare system was assessed using item C11, which asked respondents, “How much do you trust health care system (for example, hospitals, pharmacies, and other organizations involved in health care)? Responses were rated on a 4-point scale from “not at all” to “a lot.”

Physician communication quality was assessed using items C4a through C4g—“The following questions are about your communication with all doctors, nurses, or other health professionals you saw during the past 12 months”. Responses were rated on a 4-point scale from “never” to “always.” Items were averaged into a composite score, with higher scores indicating better physician communication quality.

Digital literacy was assessed using items B5a through B5c, which asked respondents to rate their agreement with statements about their comfort and confidence using technology and finding health information online, rated on a 4-point scale from “strongly disagree” to “strongly agree.” Items were averaged into a composite score, with higher scores indicating greater digital literacy.

### 2.3. Statistical Analysis

All analyses were conducted using R (version 4.5.3; R Core Team (Vienna, Austria) 2024) with the survey package to account for the complex sampling design of HINTS-7. Jackknife replicate survey weights, provided directly by NCI in the HINTS-7 public dataset, were applied to produce nationally representative estimates and valid standard errors.

Prior to regression modeling, key assumptions were examined. Multicollinearity among predictors was assessed using variance inflation factors (VIF); all VIF values were below 2.0 across both Model 2 and Model 3, indicating no multicollinearity concerns. Influential observations were examined using Cook’s distance with a threshold of 4/*n*. Approximately 4.7% of observations exceeded this threshold in the primary model, with a maximum Cook’s D of 0.009, well below the conventional cutoff of 1.0. These observations were not systematically concentrated in any demographic subgroup, and no observations were removed from the analysis.

Weighted descriptive statistics were calculated to characterize the study sample and compare wearable device use between adults with and without a history of cancer. Continuous variables are presented as weighted means with standard errors (SE), and categorical variables are presented as weighted percentages. Responses coded as −7 (missing data, question never seen) or −9 (missing data, not ascertained) were treated as missing and excluded from all analyses. Complete case analysis was used, retaining only respondents with valid data on all variables included in each model. Given the modest level of missingness and the use of survey weights, this approach was considered appropriate; however, potential bias due to missing data cannot be excluded.

Among cancer survivors who reported wearable device use, weighted descriptive statistics were used to estimate the proportion willing to share wearable-generated health data with providers and the proportion who had done so. Due to the limited number of cancer survivor wearable users with valid data on actual sharing behavior (*n* = 98), actual sharing was examined only descriptively, and these estimates should be interpreted with caution given the restricted sample size, which limits the reliability of weighted population-level generalization for this specific outcome. Survey-weighted logistic regression examined predictors of willingness to share, including trust in the healthcare system, physician communication quality, digital literacy, age, sex, education, and income. Given that willingness was the modeled outcome rather than actual sharing, findings from this model should be interpreted as describing the correlates of sharing intention rather than sharing behavior.

Survey-weighted logistic regression models were used to examine factors associated with wearable device use. Cancer survivorship status was the primary predictor of interest, with age, sex, education, income, and depression/anxiety diagnosis included as covariates to adjust for potential confounding.

Physical activity behaviors were then compared between wearable users and non-users among cancer survivors using weighted descriptive statistics. Outcomes included weekly minutes of moderate physical activity, strength training frequency, daily sitting time, and the proportion meeting aerobic physical activity guidelines (≥150 min per week).

To examine whether wearable device use was associated with meeting aerobic physical activity guidelines, three survey-weighted logistic regression models were estimated using the full adult analytic sample, which included both cancer survivors and adults without a history of cancer. This full-sample approach was necessary to support the cancer survivorship × wearable use interaction term. Model 1 was unadjusted, examining only the main effects of wearable use, cancer survivorship status, and their interaction. Model 2, the primary adjusted model, additionally controlled for age, sex, education, and household income; these variables were included as potential confounders based on prior literature demonstrating associations with both wearable use and physical activity [18,23]. Model 3 was a sensitivity analysis that further added BMI, general health, self-rated ability to manage health, mobility limitation, and pain limitation. These health-related variables may be associated with both wearable use and physical activity and were therefore included as additional covariates in a sensitivity model.

An interaction term between wearable device use and cancer survivorship status was included in all three models to test whether the association between wearable use and meeting aerobic physical activity guidelines differed by cancer survivorship status. Given the cross-sectional design of HINTS-7, all models were specified to estimate associations rather than causal effects, and findings should be interpreted accordingly. Statistical significance was defined as *p* < 0.05, and results are presented as odds ratios (OR) with 95% confidence intervals (CI).

The analytic sample varied by outcome due to item-level missingness and survey skip patterns. The full adult sample was used for wearable adoption and physical activity models to support inclusion of the cancer survivorship × wearable use interaction term. Analyses of data sharing were restricted to respondents who reported wearable device use, with actual sharing estimates further limited to those with valid responses for that item (*n* = 98). All sample sizes are reported within each analysis to ensure transparency and interpretability.

## 3. Results

### 3.1. Sample Characteristics

Among U.S. adults in HINTS-7, 34.0% of cancer survivors reported using a wearable device, compared with 41.4% of adults without a cancer history. A total of 1073 cancer survivors were identified.

Among cancer survivors, wearable users were younger than non-users. The weighted mean age among wearable users was 59.7 ± 1.4 years compared with 69.1 ± 0.9 years among non-users. The sex distribution was similar between groups, with approximately 44–46% female and 54–56% male participants (Table 1).

Wearable users generally had higher income than non-users. Among non-users, 35.3% reported annual household incomes below $35,000 compared with 19.3% of wearable users. In contrast, 40.8% of wearable users reported incomes between $75,000 and $199,999, compared with 29.5% of non-users.

### 3.2. Wearable Device Adoption Among Cancer Survivors

Survey-weighted logistic regression models examined factors associated with wearable device adoption. After adjusting for all covariates, cancer survivorship status was not significantly associated with wearable device use (OR = 1.01, 95% CI: 0.77–1.34, *p* = 0.918), nor was depression or anxiety diagnosis (OR = 1.00, 95% CI: 0.77–1.29, *p* = 0.973). Older age was associated with marginally lower odds of wearable adoption (OR = 0.98 per year, 95% CI: 0.97–0.99, *p* < 0.001). Male participants had higher odds of using wearables than females (OR = 1.35, 95% CI: 1.08–1.68, *p* = 0.009). Higher educational attainment (OR = 1.23, 95% CI: 1.14–1.33, *p* < 0.001) and higher household income (OR = 1.11, 95% CI: 1.06–1.15, *p* < 0.001) were each independently associated with greater odds of wearable device adoption.

### 3.3. Physical Activity Behaviors by Wearable Use

Cancer survivors who reported using wearable devices accumulated higher levels of physical activity compared with non-users. Wearable users reported an average of 235.70 min per week of moderate physical activity, compared with 150.20 min per week among non-users, representing an average difference of 85.50 min per week. Wearable users also reported more frequent participation in strength training (2.14 vs. 1.18 days per week). Average daily sitting time was similar between groups (6.65 vs. 6.88 h per day).

A greater proportion of wearable users met moderate aerobic physical activity recommendations compared with non-users (51.62% vs. 30.59%; Table 2). These analyses were restricted to cancer survivors with non-missing moderate physical activity data (*n* = 563).

### 3.4. Association Between Wearable Device Use and Meeting Physical Activity Guidelines

Survey-weighted logistic regression models were estimated in the full adult sample, including both cancer survivors and adults without a cancer history, to examine the association between wearable device use and meeting aerobic physical activity guidelines and to test whether this association differed by cancer survivorship status (Table 3). This full-sample approach was necessary to support the cancer survivorship × wearable use interaction term. In the unadjusted model (Model 1; *n* = 6515), wearable device use was associated with significantly higher odds of meeting physical activity guidelines (OR = 1.53, 95% CI: 1.29–1.83, *p* < 0.001). After adjusting for age, sex, education, and income in the primary model (Model 2; *n* = 6084), this association remained significant (OR = 1.42, 95% CI: 1.16–1.73, *p* = 0.001). Male sex (OR = 1.57, 95% CI: 1.27–1.94, *p* < 0.001) and higher income (OR = 1.06, 95% CI: 1.00–1.11, *p* = 0.043) were also independently associated with meeting physical activity guidelines.

The interaction term between wearable device use and cancer survivorship status was statistically significant across all four models (Model 1: OR = 1.79, 95% CI: 1.14–2.80, *p* = 0.013; Model 2: OR = 1.97, 95% CI: 1.18–3.27, *p* = 0.010; Model 3: OR = 1.89, 95% CI: 1.16–3.07, *p* = 0.012; Model 4: OR = 1.89, 95% CI: 1.18–3.02, *p* = 0.008), indicating that the association between wearable device use and meeting aerobic physical activity guidelines was consistently stronger among cancer survivors than among adults without a history of cancer.

Results from the sensitivity model (Model 4; *n* = 5907), which additionally controlled for BMI, general health, self-rated health management confidence, mobility limitation, and pain limitation, were consistent with the primary model.

### 3.5. Wearable Data Sharing and Clinical Integration

Among cancer survivors who reported wearable device use, actual shareable data was available for 98 respondents. Given the small subsample size, these estimates should be interpreted descriptively and with caution, as they may not reliably generalize to the broader population of cancer survivor wearable users. Of these, 35.4% reported having shared wearable data with a healthcare provider, 60.2% reported not sharing, and 3.6% were unsure.

For context, 77.5% of wearable users reported willingness to share device-generated data, resulting in an approximate 42 percentage-point difference between willingness and reported sharing behavior.

Survey-weighted logistic regression models examined predictors of willingness to share wearable data. No measured patient-level covariates—including trust in the healthcare system, physician communication quality, digital literacy, age, sex, education, or income—were significantly associated with willingness to share (all *p* > 0.05). This may reflect uniformly high willingness across subgroups as well as limited statistical power (*n* = 294).

Because valid data on actual sharing behavior were available for a limited subsample, analyses of sharing were descriptive, and no regression model was estimated for actual sharing.

## 4. Discussion

The present study used nationally representative HINTS-7 data to examine wearable device adoption, physical activity behaviors, and wearable-generated data sharing among U.S. adults, with particular attention to cancer survivors. Findings are interpreted as population-level estimates of associations and behaviors rather than causal effects, given the cross-sectional design.

Wearable device use was defined as any use within the past 12 months, providing a standardized exposure that reflects general adoption rather than short-term engagement. Using this definition, wearable adoption was common in the U.S. adult population but differed across demographic groups. Younger age, male sex, higher education, and higher income were associated with wearable use, whereas cancer survivorship status was not. These findings position wearable adoption among cancer survivors within the broader population distribution, rather than as a distinct pattern.

### 4.1. Wearable Device Adoption Among Cancer Survivors

The results indicate that cancer survivorship status itself was not independently associated with wearable device adoption after adjusting for sociodemographic factors. Instead, age, sex, education, and household income emerged as the primary predictors of wearable use, suggesting that barriers to wearable adoption among cancer survivors are likely driven more by broader socioeconomic characteristics than by survivorship-related factors alone.

This pattern is consistent with prior research demonstrating that digital health technology adoption tends to mirror existing socioeconomic disparities in access to technology and health resources [18,26], In the present study, higher educational attainment and income were associated with greater wearable adoption, reflecting broader patterns in which individuals with more socioeconomic resources and digital access are more likely to engage with wearable technologies. Older adults were less likely to adopt wearable devices, which may reflect differences in digital literacy, perceived usefulness, or usability barriers. That these same disparities appear among cancer survivors is notable: older survivors and those with fewer economic resources often face greater health burdens, including higher rates of comorbidity, functional limitation, and reduced access to structured exercise programs, and may therefore stand to benefit most from accessible tools that support physical activity monitoring and behavioral engagement [4].

These findings suggest that wearable adoption within survivorship care should be considered not only a behavioral issue but also a potential health equity consideration at the population level, as adoption patterns reflect broader socioeconomic disparities in access to digital health tools.

### 4.2. Wearable Device Use and Physical Activity

A central finding of this study was a significant interaction between wearable device use and cancer survivorship status in predicting adherence to aerobic physical activity guidelines. In the primary adjusted model, wearable device use was associated with higher odds of meeting physical activity guidelines, and this association was significantly stronger among cancer survivors than among adults without a history of cancer.

Given the cross-sectional design, this pattern should be interpreted as a difference in association rather than evidence of a differential effect of wearable use. One possibility is that wearable technologies provide behavioral support through real-time feedback, goal tracking, and activity monitoring, which are consistent with established behavior change frameworks such as self-regulation theory and Social Cognitive Theory, where self-monitoring and feedback are key mechanisms supporting behavior engagement [27,28]. These features may be heightened following a cancer diagnosis, a phenomenon described in the literature as a “teachable moment” [28].

An equally plausible alternative, however, is that cancer survivors who are already more physically active or health-motivated are more likely to adopt wearable devices, such that the observed association reflects selection rather than a device effect. This is consistent with models of health behavior that emphasize pre-existing motivation and readiness to change as drivers of both technology adoption and engagement in physical activity [18,27]. Both behaviors may also reflect unmeasured underlying health motivation.

Longitudinal and intervention-based research will be necessary to distinguish between these explanations and determine whether wearable device use leads to sustained increases in physical activity among cancer survivors. These findings align with theoretical perspectives suggesting that wearable technologies may function as facilitators of behavior among already motivated individuals, while also highlighting the need to examine how such tools operate across different levels of readiness and health status.

These findings document a cross-sectional co-occurrence pattern: wearable use and physical activity guideline adherence are more strongly associated among cancer survivors than among the broader adult population at the national level. This observation, which could reflect device-related support, selection, shared health motivation, or some combination, provides a descriptive benchmark rather than evidence of a causal benefit. It provides a population-level reference point for future longitudinal and intervention-based studies examining wearable use within survivorship care, particularly given the well-documented barriers survivors face in sustaining physical activity.

Several health-related factors were independently associated with lower odds of meeting physical activity guidelines in the sensitivity model, including higher BMI, poorer general health, and lower confidence in managing personal health. These patterns suggest that wearable use occurs within a broader context of health status and behavioral capacity, and that additional supports beyond device use may be relevant for survivors with greater health burdens.

### 4.3. Wearable Data Sharing and Clinical Integration

Wearable devices can generate continuous streams of patient-generated health data with direct relevance for survivorship care. Recent work suggests that wearable-derived activity data may support remote monitoring, early identification of functional decline, and personalized care outside traditional clinical settings [29,30,31].

Despite this potential, the present study identified a substantial descriptive gap between patient willingness and actual data-sharing behavior. Among cancer survivors using wearable devices, 77.5% expressed willingness to share device-generated data with their healthcare provider, yet only 35.4% of those with valid actual sharing data (*n* = 98) reported having done so. These nationally representative estimates provide a quantitative benchmark for the differences between intention and observed behavior in wearable data sharing among cancer survivors.

This discrepancy raises questions regarding the factors that influence whether wearable data are ultimately shared in clinical contexts. In the current study, no measured patient-level predictors, including trust in the healthcare system, physician communication quality, digital literacy, age, sex, education, or income, were significantly associated with willingness to share wearable data. This null finding is consistent with uniformly high willingness across patient subgroups and with limited statistical power and should not be interpreted as evidence that no patient-level predictors exist.

Because actual sharing, rather than willingness, could not be modeled as an outcome due to insufficient sample size, strong conclusions about the locus of the implementation barrier cannot be drawn from this study alone. However, the observed pattern, high willingness paired with lower reported sharing, does not appear to be explained by measured patient-level characteristics and warrants further investigation.

Recent research in digital health highlights several potential barriers to sharing patient-generated data, including concerns about privacy, data security, and unclear data governance, as well as limited interoperability between wearable platforms and electronic health record systems [32,33]. Additionally, both patients and clinicians have reported uncertainty regarding how wearable data should be interpreted and incorporated into care, which may limit its practical use in clinical settings [34,35].

If structural or system-level barriers are confirmed in future research, improving clinical integration of wearable-generated data may require health system changes, including standardized data collection workflows, electronic health record integration pathways, and clinical guidance for interpreting wearable-derived activity data. These findings should be interpreted as identifying a measurable gap rather than establishing its underlying cause.

These population-level estimates provide nationally representative benchmarks for wearable device use, physical activity behaviors, and data sharing among cancer survivors, and can serve as a reference point for future intervention studies and health system quality improvement efforts [36]. The observed gap between willingness to share and actual data sharing, which was not explained by measured patient-level characteristics, highlights a measurable clinical integration gap and represents a critical target for future research and health system innovation aimed at incorporating patient-generated health data into survivorship care.

### 4.4. Limitations

Several limitations should be considered when interpreting these findings. First, the cross-sectional design of HINTS-7 precludes causal inference, making it impossible to determine whether wearable device use leads to increased physical activity or whether individuals who are already more active are more likely to adopt wearable technologies. Second, all variables, including physical activity, wearable device use, and health status, were based on self-reported survey responses [37], which may introduce recall and social desirability bias [38]. Third, the analytic sample of cancer survivors with wearable device use who had complete data for the data-sharing models was relatively modest (*n* = 294), which limited statistical power for the willingness-to-share analyses and should be considered when interpreting the null findings in Table 4. Fourth, item-level missingness across covariates resulted in modestly reduced analytic sample sizes across regression models, ranging from *n* = 6515 in the unadjusted model to *n* = 5907 in the fully adjusted sensitivity model, and complete case analysis was used throughout. Only 11 of 1073 cancer survivors (1.0%) had genuinely missing wearable status and were excluded from wearable-related analyses; the remaining 1062 provided valid responses and were retained. Fifth, the HINTS-7 data-sharing items assess willingness to share wearable-generated health data with a healthcare provider broadly, without specifying what type of data or provider; as such, our analyses cannot characterize what specific data cancer survivors intended to share or whether sharing was directed toward oncology care specifically. Finally, although HINTS-7 includes clinical survivorship variables such as cancer type, stage of diagnosis, and family history of cancer, our analysis did not stratify analyses by these characteristics. Given the relatively small subsample of cancer survivors reporting wearable device use (*n* = 326), stratifying by cancer type would have produced unstable estimates. Future research using larger cancer survivor samples or registry-linked samples would allow for more nuanced examination of how wearable adoption and physical activity behaviors vary across cancer types and clinical survivorship trajectories.

## 5. Conclusions

This study provides nationally representative estimates of wearable device adoption, physical activity behaviors, and data sharing among U.S. cancer survivors. Wearable use and meeting aerobic physical activity guidelines showed a stronger relationship among cancer survivors than among adults without a cancer history, although causality cannot be inferred. Disparities in wearable adoption by age and socioeconomic factors persist, and a substantial gap between willingness to share wearable data and actual sharing highlights a critical barrier to clinical integration.

## Figures and Tables

**Table 1:** Demographics of Cancer Survivors by Wearable Use (*n* = 1062).

Variables	No Wearables (*n* = 732)	Wearables (*n* = 330)
Age (years; mean ± SE)	65.6 ± 1.13	62.6 ± 0.94
Female (%)	43.2	40.4
Male (%)	56.8	59.6
BMI (kg/m^2^; mean ± SE)	27.9 ± 0.47	27.4 ± 0.45
Functional limitations (%)	41.8	35.2
Education (%)		
Less than high school	3.1	0.6
High school graduate	5.9	2.1
Some college	14.3	11.8
Associate degree	12.2	4.9
Bachelor’s	23.3	22.4
Some graduate	19.9	30.0
Graduate degree	19.5	27.0
Income (%)		
<$35,000	35.3	19.3
$35,000–74,000	25.2	27.3
$75,000–199,000	29.5	40.8
≥$200,000	10.1	12.5
General Health (%)		
Excellent	6.3	9.4
Very good	22.0	33.3
Good	39.4	39.1
Fair	26.8	14.8
Poor	4.9	3.0

Note: Data are from the Health Information National Trends Survey (HINTS-7, 2024). Values reflect survey-weighted estimates. Age and BMI are presented as mean ± standard error (SE). All other values are weighted percentages. BMI = body mass index; kg = kilogram; m = meters; % = percentage.

**Table 2:** Physical Activity Behaviors Among Cancer Survivors by Wearable Use (*n* = 1039).

Outcome	No Wearable Mean (*n* = 713)	Wearables Mean (*n* = 326)
Weekly moderate PA (minutes/week)	150.20 (15.28)	235.70 (15.85)
Strength training (days/week)	1.18 (0.14)	2.14 (0.25)
Sitting time (hours/day)	6.88 (0.22)	6.65 (0.38)
Meeting aerobic PA guideline (%)	30.59	51.62

Note: Values are mean (SE) unless otherwise indicated. Aerobic PA guideline is defined as ≥150 min per week of moderate-intensity physical activity, consistent with ACS Guidelines recommendations. Analyses restricted to cancer survivors with non-missing moderate physical activity data. PA = physical activity; SE = standard error; % = percentage.

**Table 3:** Survey-Weighted Logistic Regression Predicting Meeting Aerobic Physical Activity Guidelines (≥150 min/week) Among All U.S. Adults (Cancer Survivors and Non-Cancer Adults Combined; Full Analytic Sample).

Predictors	Model 1 (Unadjusted) *n* = 6515	Model 2 (+Age, Sex) *n* = 6390	Model 3 (+Education, Income) *n* = 6084	Model 4 (+Health Status) *n* = 5907
	OR (95% CI) *p*	OR (95% CI) *p*	OR (95% CI) *p*	OR (95% CI) *p*
Wearable use (vs. no)	1.53 (1.29–1.82) *p* < 0.001 *	1.55 (1.31–1.85) *p* < 0.001 *	1.42 (1.17–1.72) *p* < 0.001 *	1.50 (1.22–1.85) *p* < 0.001 *
Cancer survivor (vs. no cancer)	0.76 (0.57–1.03) *p* = 0.075	0.79 (0.58–1.09) *p* = 0.151	0.73 (0.52–1.02) *p* = 0.065	0.76 (0.54–1.07) *p* = 0.117
Wearable use × Cancer survivor	1.79 (1.15–2.77) *p* = 0.010 *	1.78 (1.11–2.86) *p* = 0.017 *	1.97 (1.20–3.22) *p* = 0.007 *	1.89 (1.18–3.02) *p* = 0.008 *
Age (years)	—	1.00 (0.99–1.00) *p* = 0.382	1.00 (0.99–1.00) *p* = 0.442	1.00 (1.00–1.01) *p* = 0.621
Female (vs. Male)	—	1.64 (1.34–2.00) *p* < 0.001 *	1.57 (1.28–1.93) *p* < 0.001 *	1.52 (1.22–1.91) *p* < 0.001 *
Education	—	—	1.03 (0.97–1.10) *p* = 0.355	1.00 (0.93–1.07) *p* = 0.959
Income	—	—	1.06 (1.00–1.11) *p* = 0.037 *	1.01 (0.96–1.06) *p* = 0.762
BMI (kg/m^2^)	—	—	—	0.98 (0.96–0.99) *p* = 0.001 *
General health	—	—	—	1.37 (1.20–1.56) *p* < 0.001 *
Health self-efficacy	—	—	—	1.28 (1.11–1.47) *p* = 0.001 *
Mobility limitation	—	—	—	0.87 (0.58–1.29) *p* = 0.477
Pain limitation	—	—	—	1.02 (0.74–1.41) *p* = 0.908

Note: OR = odds ratio; CI = confidence interval; kg = kilograms; m = meters; BMI = body mass index. * *p* < 0.05. Model 2 is the primary adjusted model. Model 3 is a sensitivity analysis; health status variables (BMI, general health, health management confidence) may partially lie on the causal pathway between cancer survivorship, wearable use, and physical activity and are therefore presented separately. Model 4 additionally controls for BMI, general health, self-rated health management confidence, mobility limitation, and pain limitation.

**Table 4:** Predictors of Willingness to Share Wearable Data (*n* = 294).

Predictors	OR	95% CI	*p*
Trust in healthcare system	0.45	0.14–1.46	0.178
Physician communication quality	0.99	0.96–1.02	0.556
Digital literacy	1.01	0.98–1.04	0.383
Age (years)	1.02	0.99–1.05	0.244
Male (vs. Female)	1.74	0.45–6.66	0.410
Education	1.09	0.68–1.76	0.706
Income	0.98	0.72–1.34	0.903

Note: OR = odds ratio; CI = confidence interval. Model estimated using survey-weighted logistic regression with jackknife replicate weights. The dependent variable is willingness to share wearable-generated health data with a healthcare provider (yes vs. no). No predictors were statistically significant at *p* < 0.05, indicating that willingness was uniformly high across patient subgroups and was not explained by measured attitudinal, demographic, or socioeconomic characteristics.

## Data Availability

The HINTS-7 dataset is publicly available at no cost through the National Cancer Institute at https://hints.cancer.gov/data/download-data.aspx (accessed on 1 March 2026).

## Abbreviations

The following abbreviations are used in this manuscript:

ACS	American Cancer Society
BMI	Body mass index
CA	Cancer (journal abbreviation)
CI	Confidence interval
CS	Cancer survivors
HINTS	Health Information National Trends Survey
HINTS-7	Health Information National Trends Survey, Cycle 7
HCP	Healthcare provider
NCI	National Cancer Institute
OR	Odds ratio
PA	Physical activity
SE	Standard error
U.S.	United States
VIF	Variance inflation factor
